# Biomarkers in Acute Coronary Syndrome

**DOI:** 10.4137/bmi.s588

**Published:** 2008-11-05

**Authors:** Valentina Loria, Milena Leo, Gina Biasillo, Ilaria Dato, Luigi M. Biasucci

**Affiliations:** Institute of Cardiology Catholic University 8 Largo Gemelli 00168 Rome, Italy

## Abstract

**Background:**

Evaluation of patients who present to the hospital with acute undifferentiated chest pain or other symptoms and signs suggestive of Acute Coronary Syndrome (ACS) is often a clinical challenge.

The initial assessment, requiring a focused history (including risk factors analysis), a physical examination, an electrocardiogram (EKG) and serum cardiac marker determination, is time-consuming and troublesome. Recent investigations have indicated that increases in biomarkers of necrosis, inflammation, ischemia and myocardial stretch may provide earlier assessment of overall patient risk, help in identifying the adequate diagnostic and therapeutic management for each patient and allow for prevention of substantial numbers of new events.

**Approach and Content:**

The purpose of this review is to provide an overview of the characteristics of several biomarkers that may have potential clinical utility to identify ACS patients. Patho-physiology, analytical and clinical characteristics have been evaluated for each marker, underlying the properties for potential routine clinical use.

**Summary:**

The biomarkers discussed in this review are promising and might lead to improved diagnosis and risk stratification of patients with ACS, however their clinical application requires further studies. It is important to define their clinical role as diagnostic markers, their predictive value and the specificity, standardization and detection limits of the assays.

## Introduction

The term “acute coronary syndrome” (ACS) encompasses a range of thrombotic coronary artery diseases, including unstable angina (UA) and both ST-segment elevation (STEMI) and non-ST-segment elevation myocardial infarction (NSTEMI).

Current estimates are that 1.7 million patients with ACS are admitted each year to hospitals in the United States (American Heart Association: 2004 Heart and Stroke Statistical Update). Of these, only one-quarter present with STEMI; three quarter, or approximately 1.4 million patients, have UA or NSTEMI. It’s further estimated that 4% of patients admitted to the Emergency Department (ED) with Acute Myocardial Infarction (AMI) have an inadequate discharge with considerable risk of cardiac events at home for patients (Lee TH et al. 1987).

It is evident that the epidemiological importance of the phenomenon, the potentially lethal consequences for the patient, and the economical and legal implications for clinicians make the need of an adequate strategy of diagnostic and therapeutic management, including risk stratification and prevention of possible new events.

However, the definition of the assessment protocol in the evaluation of patients with chest pain or other symptoms suspected for ACS is difficult. ACS requires early identification, adequate risk stratification and management: patients with ongoing chest pain and persistent ST-segment elevation (or new-onset left bundle branch block) require immediately recanalization by fibrinolytic treatment or primary angioplasty in patients with chest pain and EKG abnormalities suggesting acute ischemic heart disease, the strategy is to value the likelihood of ACS and to confirm or rule out myocardial necrosis, to alleviate ischemia and symptoms, to observe with serial EKG, to repeat measurements of markers of myocardial necrosis and to initiate appropriate therapy. However, suggested approach consistent of history, EKG and serum cardiac markers determination is time-consuming and not necessarily accurate: clinical presentation of ACS is often atypical, EKG abnormalities are not always present (Pope JH et al. 1998) and markers of myocardial necrosis may be negative at admission.

Recent investigations have indicated that increases in several biomarkers upstream may provide earlier assessment of overall patient risk, help in identifying the adequate diagnostic and therapeutic management for each patient and allow for prevention of cardiac new events. It is now apparent that ACS shares a common anatomical substrate: pathological, angioscopic and biological observations have demonstrated that UA and acute myocardial infarction (AMI) are different clinical presentations that result from a common underlying patho-physiological mechanism, namely atherosclerotic plaque rupture or erosion, with different degrees of superimposed thrombosis and distal embolization (Davies MJ et al. 1993; Davies M, 1995; Davies M, 1997).

## Traditional Markers of Myocardial Damage

### Creatine kinase (CK) and creatine kinase-MB (CK-MB)

Creatine kinase (CK) and creatine kinase-MB (CK-MB) have a long history as the gold standard for AMI diagnosis. CK-MB is a CK isoenzyme, predominantly found in the myocardium. Its elevation occurs 4–6 h after the onset of myocardial necrosis and remains for 24–48 h. CK-MB sensitivity and specificity in detecting myocardial injury can be increased by serial testing (Gibler WB et al. 1990). CK-MB is relatively sensitive, but its specificity is affected by the presence of this marker in skeletal muscle. Elevation in CK-MB, in fact, may occur as a result of occasional analytical interferences and in patients with trauma, rabdomyolysis, myopathies, renal failure or during the peripartum period. To improve its specificity, it was proposed to use CK-MB relative index (CK-MB/total CK). Ratios greater than 2.5 percent are considered suggestive of myocardial damage (Pearson JR et al. 1990). Mass assays for CK-MB have largely replaced older activity assays based on electrophoresis, and because of their better analytical sensitivity and precision. CK-MB activity assay were and are limited by their inability in detecting low concentrations of CK-MB. In addition, electrophoresis and column chromatography methods were subject to false-positive interference from so-called macro-CK, caused by an immune response against CK-MB in some populations. For these reasons, CK-MB activity assays gave away to more analytically sensitive and specific mass immunoassays that offered the advantage of quantifying CK-MB as a specific protein, rather than relying on functional enzymatic activity as a surrogate. CK-MB mass, CK-MB activity and total CK are more specific than myoglobin, but may not be detectable for 4–6 h in the bloodstream following myocardial injury. The sensitivity of CK-MB mass for AMI is only 50% when measured early at the time of presentation (Bertrand ME et al. 2002). In clinical practice, peak levels of markers of necrosis and area under the time release curve of CK-MB from repetitive serial samplings are used to estimate infarct size.

### Cardiac troponin T (cTnT) and I (cTnI)

Cardiac troponin T (cTnT) and cardiac troponin I (cTnI) are more sensitive and specific markers than CK-MB in detecting myocardial necrosis, and have become the preferred biomarkers for the diagnosis of AMI. They are also a useful prognostic indicator in patients with ACS. The superior clinical performance of troponin results from its higher sensitivity for smaller myocardial injury and its virtually total specificity for cardiac damage (Jaffe AS et al. 2000) and is considered the gold-standard in the new definition of myocardial infarction (Thygesen et al. 2007). Troponin is a complex of three proteins that is integral to muscle contraction in skeletal and cardiac muscle, regulating the calcium-mediated interaction between actin and myosin. Its three subunits are TnC, TnI, and TnT. Troponin C binds to calcium ions in order to produce movement; Troponin T binds to tropomyosin, interlocking it to form a troponin-tropomyosin complex; Troponin I binds to actin in thin myofilaments to hold the troponin-tropomyosin complex in place. When calcium is bound to specific sites on TnC, the structure of the thin filament changes in such a manner that myosin (a molecular motor organized in muscle thick filaments) attaches to thin filaments and produces force and/or movement (Gillis TE et al. 2007). In the absence of calcium, tropomyosin interferes with action of myosin, and therefore muscles remain relaxed. Troponin C has an identical amino-acid sequence in both skeletal and cardiac tissues and, thus, it has no potential as a cardiac specific marker. However Troponin T and Troponin I have different isoforms in cardiac and skeletal muscle, encoded by separated genes, and consequently, have different amino-acid sequences (Christenson RH and Azzazy HME, 1998). The respective cardiac isoforms of TnT (cTnT) and TnI (cTnI) allow production of antibodies that exclusively recognize these myocardial-specific proteins. To date, cTnT and cTnI release has not been attributed to a tissue source other than myocardium, and except for rare analytical false positives, detection of cTnT or cTnI in the blood is indicative of heart injury (Jaffe AS, 2001). Serial measurements of cardiac troponin significantly improve the ability of this biomarker in detecting AMI (REACTT Investigators study group 1997). Hamm et al. (Hamm et al. 1997) performed troponin testing upon presentation and at 4 h with improvement in the sensitivity from 51% to 94% for cTnT and 66% to 100% for cTnI in patients without ST-elevation. Higher diagnostic sensitivity and specificity require specimen collection at patient presentation, 6–9 h later and at 12–24 h if clinical suspicion is high and earlier results are negative. Indeed, troponin is not considered as an early biomarker of myocardial necrosis: cardiac troponins need 4–10 h after symptoms onset to appear in serum, and peak at 12–48 h, remaining then abnormal for several days to two weeks (Panteghini M et al. 1999). However, troponins can help to identify such patients at high risk of infarction and death for whom earlier and more invasive investigation is indicated. Increase in troponin levels represents a powerful marker of high short-term risk of death or nonfatal myocardial infarction in patients with non-ST-elevation acute coronary syndromes (NSTE-ACS); it is one of the indicators for an early invasive strategy, according to American College of Cardiology/American Heart Association guidelines (ACC/AHA) (Anderson JF et al. 2007). There is also a relationship between the severity of the infarct and the duration of the elevated serum cardiac troponins. The release periods of troponin in patients with NSTEMI are significantly less than those with ST-elevation at EKG, and troponin elevations in traditionally defined unstable angina patients, representing microscopic infarct, might last only several hours at a time (Panteghini et al. 2002). There is substantial evidence that 30% of patients with unstable angina have circulating troponins, indicating a subset of patients with cell damage (Collinson PO et al. 1996). When troponin elevations occurred without elevation of CK or CK-MB, patients were often classified as having “minor myocardial damage”, yet they have increased subsequent frequencies of death, myocardial infarction and need for revascularization (Ohman EM et al. 1996; Olatidoye, 1998) and therefore are now considered as myocardial infarction. From a clinical perspective, there is evidence that any amount of detectable cardiac troponin release is associated with an increased risk of new adverse cardiac events (Kavsak et al. 2007). The new global definition of myocardial infarction receives and organizes these observations defining any amount of cardiac troponin >99th percentile (10% Coefficient of Variation method) as myocardial infarction. Haft and colleagues (Haft JI and Saadeh SA, 1997) reported that patients with elevated troponin values tend to have a high incidence of complex lesions and that these lesions tend to be of very high grade. In contrast, individuals without elevations of troponin are less likely to have complex lesions and in general have lesions that are less severe in terms of the percentage of obstruction. The role of troponins as a tool for identification of patients at high risk is of interest also for therapeutic management. In a retrospective analysis, Hamm (Hamm CW et al. 1999) segregated patients in two groups by the presence or absence of increased plasmatic levels of cTnT and demonstrated that only individuals with increased levels of cTnT benefit from abciximab therapy, with substantially reduced events rate both at 30 days and at 6 months. In contrast, in the group without elevations of cTnT, abciximab had no statistically significant effect on event rates. Thus, evaluation of troponin values may provide a strategy for the therapeutic subsetting of patients with unstable angina and those in need of angioplasty procedures who may be at risk for complications. It appears that patients with elevations of the troponins may require more aggressive anticoagulant/antithrombotic therapy than those with normal values (Lindahl B et al. 1997).

These data demonstrate that troponin is a powerful marker for the hallmark of AMI and a useful tool in assessing risk and directing appropriate therapy that improves clinical outcome. Although cardiac troponins are specific of cardiac damage, they cannot be considered as the definitive marker of myocardial ischemic injury. Troponin release occurs due to non-ischemic cardiac damage, too, causing “false positive” elevations: myopericarditis, aortic dissection, aortic valve disease, hypertrophic cardiomyopathy, cardiac contusion or other trauma, tachy- or bradyarrhytmias, apical ballooning syndrome, congestive heart failure. Furthermore, elevation of cardiac troponin can also occur in non primary cardiac pathologies, such as acute neurological diseases, including stroke or sub-arachnoid hemmorrhage, infiltrative diseases, drug toxicity or toxins, burns affecting >30% of body surface area (Thygesen K et al. 2007). An other important clinical challenge is the significance of elevated concentrations of cTnT commonly found in patients with renal failure but no clinical signs of recent myocardial damage (Freda BJ et al. 2002); even in this setting, however, raised levels of troponin are associated with adverse cardiac prognosis.

There are a few methodological problems, in particular the lack of standardisation of assays for cTnI. Plasma levels of cTnI are associated with cardiac morbidity and mortality and have an independent prognostic value in patients with ACS (Galvani M et al. 1997) but there is no consensus on common reference values and standardization between the several commercially available cTnI assays. More than 15 companies presently market assays for cTnI employing different standard materials and antibodies with different epitope specificities. Consequently, different results from different cTnI systems and assay generations may be obtained and this problem may cloud the interpretations of reported data, creating a substantial problem for the clinical and laboratory communities (Apple FS, 1999).

### B-type natriuretic peptide (BNP)

B-type natriuretic peptide (BNP) is preferentially produced and secreted in the left ventricle, although the right side of the human heart also synthesises and secretes BNP in response to disease (Kay JD et al. 2003). The predominant stimulus controlling the synthesis and release of BNP from cardiac atria and ventricles is wall stretch. BNP expression and release from cardiomyocites can also be stimulated by a variety of endocrine, paracrine and autocrine factors that are activated in heart failure, including norepinephrine, angiotensin II, ET-1, glucocorticoids and proinflammatory cytokines. In general, the plasma concentrations of these peptides are increased in diseases characterised by an expanded fluid volume, such as renal failure, primary aldosteronism and congestive heart failure (CHF), or by stimulation of peptide production caused by ventricular hypertrophy or strain, thyroid disease, excessive circulating glucocorticoids or hypoxia (Clerico A et al. 1999). Natriuretic peptides inhibit renin-angiotensin-aldosterone axis, increasing diuresis and reducing ventricular preload and arterial blood pressure, and inhibit central sympathetic outflow and catecholamine release from peripheral sympathetic neurons.

Available Commercial immunoassays measure BNP and its N-terminal inactive form NT-proBNP. The half-life of BNP is 20 min whereas NT-proBNP has a half-life of 120 min (Kemperman H et al. 2004), which explains why NT-proBNP serum values are approximately six times higher than BNP values, even though both molecules are released in equimolar proportions (Hall C, 2005).

Because of the longest half-life NT-proBNP could be more sensitive than BNP in the diagnosis of the earliest phase of the left ventricular dysfunction (Hunt PJ et al. 1995). Many assays, including those for the promising Mid Terminal (MT) BNP are now under development. It has been shown in several studies that BNP and NT-proBNP are related to sex, with higher values in females, and to age, with higher values in older individuals. In patients with reduced renal function, BNP and NTproBNP values are increased with a negative correlation to creatinine clearance. Worsening renal function affects clearance of NT-proBNP greater than BNP (Weber M and Hamm C, 2006), however deFilippi et al. have recently shown that the diagnostic accuracies of BNP and NT-proBNP in detecting decompensated heart failure are similar in patients with or without renal failure and that NT-proBNP is superior to BNP in predicting mortality (deFilippi CR et al. 2007).

The clinical usefulness of cardiac natriuretic peptides (especially BNP and NT-proBNP) in the evaluation of patients with suspected heart failure, in prognostic stratification of patients with CHF, in detecting LV systolic or diastolic dysfunction and in the differential diagnosis of dyspnoea was confirmed (Cowie MR et al. 2003). In general, heart failure is unlikely at BNP values <100 pg/ml and is very likely at BNP values >500 pg/ml and, similarly, unlikely at NT-proBNP values <300 pg/ml and very likely at NT-proBNP values >450 pg/ml (>900 pg/ml in patients above 50 years of age) (Kay JD et al. 2003). Independent of their diagnostic value, several large scale studies have convincingly shown that BNP and NT-proBNP provide strong prognostic information for an unfavourable outcome (death, cardiovascular death, readmission or cardiac events) in patients with heart failure or asymptomatic left ventricular dysfunction. It was also shown that a decrease in BNP during the initial hospital stay was associated with a favourable clinical outcome, whereas no change or an increase in BNP values was associated with an unfavourable outcome (Struthers AD, 1999).

Although originally BNP and NT-proBNP were considered biomarkers for heart failure only, now they are also considered biomarkers of myocardial ischemia. Elevated BNP and NT-pro-BNP levels have been observed in patients with stable coronary artery disease (CAD), in patients with UA (Kikuta K et al. 1996) and during and after Percutaneous coronary intervention (PCI) (Tateishi et al. 2000). From recent studies it has emerged that the long and short-term prognostic power of BNP and NT-proBNP is similar, in the acute myocardial infarction with ST-elevation and without ST-elevation, both at hospital admission and during hospitalization (Galvani et al. 2004). It is widely believed that the underlying patho-physiological process causing an increase in BNP and NT-proBNP values is left ventricular systolic or diastolic dysfunction caused by myocardial ischemia that leads to an increased wall stress. Nevertheless, data derived from experimental studies suggest a direct release of BNP and NTproBNP from cardiomyocytes in response to myocardial ischemia independent of ventricular wall stress (Morita E et al. 1993). In the early phase after AMI there is an augmented production of cardiac natriuretic peptides related to myocardial stretch secondary to LV dysfunction, to increased heart rate, hypoxia and ischemia per se. Recent studies suggest that the augmented release of BNP following brief periods of ischemia occurs without concomitant change in LV end diastolic pressure, suggesting that ischemia per se is the stimulus for BNP release (D’Souza SP and Baxter GF, 2003). BNP and NT-pro-BNP have also emerged as prognostic indicators of long-term mortality early after an acute coronary event. This association was observed across the spectrum of ACS, including patients with STEMI, NSTEMI and UA, those with and without elevated cardiac troponins, and those with and without clinical evidence of heart failure (de Lemos JA and Morrow DA, 2002; White HD and French JK, 2003). Substudies of large scaled clinical trials (Varo N et al. 2003; Sabatine MS et al. 2002; Lindahl B et al. 2005; Jernberg T et al. 2004; Heeschen C et al. 2004) have evaluated the prognostic value of BNP and NT-proBNP in patients presenting with NSTE-ACS. In all studies elevated values of BNP and NT-proBNP have consistently been found. In a recent analysis made by the group of Sabatine (Sabatine et al.2002)in 450 patients of the OPUS-TIMI 14 and in 1635 patients of the TACTICS-TIMI18 (de Lemos JA and Morrow DA, 2002) in which was investigated an approach with multiple markers in ACS without ST elevation, BNP, C-Reactive Protein (CRP) and cTnI were all independent predictors of adverse outcome (Sabatine et al. 2002). The incidence of the adverse events not only resulted correlated with the positivity of each marker but also with the number of positive markers. Although patients with the worse prognosis (with a relative risk of death to 30 days between 6.0 and 13.0) were those with combined increase of levels of cTnI (marker of thrombosis and myocardial necrosis), of CRP (expression of inflammatory status) and of BNP (marker of myocardial dysfunction). Similar results were reported for the predictive value of BNP and NT-proBNP after STEMI. Quite recently, plasma natriuretic peptide concentrations were also related to risk of cardiovascular events and death in apparently asymptomatic persons (Wang TJ et al. 2004).

The therapeutic benefits that can derive from BNP and NT-proBNP assessment in ACS are not clear. In a substudy of the FRISC-II trial (Lindhal B et al. 2005) which investigated the usefulness of NT-proBNP for identifying patients who might benefit from an early invasive strategy, a trend towards a better outcome of patients with NT-proBNP values in the highest tertile was observed. In the substudy of the PRISM trial, Heeschen et al. analysed the effect of glycoprotein IIb/IIIa inhibition with tirofiban with respect to NT-proBNP values (Heeschen et al. 1999). They found that patients with high NT-proBNP values had a lower event rate with tirofiban treatment compared to placebo at 48 h, they found no significant interaction between NT-proBNP values and the clinical benefit of tirofiban treatment at 30 days. Further studies are needed to assess the therapeutic benefits that can derive from BNP and NT-proBNP assessment. Recent data suggest that natriuretic peptides may help to better identify the very high risk patients and the very low risk ones among a population of ACS patients with respectively positive or negative troponin. The role of BNP and NT-proBNP testing is included in the guidelines for the diagnosis and treatment of chronic heart failure of the task force of the European Society of Cardiology, published in 2005. Assessment of both markers is considered to be a reliable rule-out test of heart failure in primary care and in the emergency room. Assessment of BNP for risk stratification is also mentioned in the guidelines of the European Society of Cardiology for management of acute coronary syndromes in patients presenting without persistent ST segment elevation, but without any recommendations for the application of BNP and NT-proBNP in clinical routine (Kay JD et al. 2003).

### C-Reactive protein (CRP)

CRP is the most widely studied inflammation marker. CRP is a pentraxin, synthesized by liver after stimulation by cytokines, especially IL-6, IL-1β and TNF-α, in response to tissue injury or infection (Volanakis JE, 2001). CRP is an acute-phase protein and a marker of inflammation, but has also an active role in the innate immune response: 1) it activates the classic complement system, 2) rules the expression of the endothelial NO synthetase and the synthesis of NO and 3) induces the expression of the molecules of adhesion and the activation of the NF-kB. Moreover, it can directly regulate the oxidation of the LDLs.

The advantages of CRP as an inflammatory biomarker are related in part to analytic properties (such as the availability of low-cost, accurate high-sensitivity assay) and in part to its biological profile, including a long half-life (19 h). Liuzzo et al. ( Liuzzo G et al. 1994) showed that patients presenting with unstable angina and elevated plasma concentrations of CRP had a higher rate of death, AMI and need for revascularization compared with patients without elevated concentrations. In more recent trials, other investigators have confirmed the increased risk associated with higher CRP concentrations (Morrow DA et al. 1998). In the majority but not in all the studies, CRP has been showed to be a marker of the short—term risk (in hospital or 30 days) of recurrent cardiac events, including, death, AMI, and urgent revascularization (Biasucci LM et al. 2000). More consistently CRP was found to be independently associated with the recurrence of cardiovascular events and with death in the mid to long term (Biasucci LM et al. 1999; Heeschen C, 2000). An incremental prognostic power of CRP along with Troponin has been clearly demonstrated (Rebuzzi AG et al. 1998; Morrow DA et al. 1998). This association is of similar magnitude to other major clinical predictors of complications in ACS, including age, ST-abnormalities and cardiac troponin. In each of the above studies, the predictive value of CRP was independent of, and additive to, cardiac troponin. More importantly, CRP was found to have prognostic value even among patients with negative cardiac troponin and no evidence of myocyte necrosis (Lindahl B et al. 2000). Therefore its prognostic relationship is not a merely consequence of the inflammatory response to myocardial necrosis and is present in patients with no evidence of myocardial injury as detected by cardiac troponin (Liuzzo G et al. 1994), although the data were obtained with troponin assays not at high sensitivity.

A variety of cut-off points have been used in clinical studies of CRP in ACS, leaving some uncertainty about the optimal decision limits. In primary prevention studies the relationship is graded with a very low risk reported for CRP levels < 1.0 mg/l, intermediate risk between 1 and 3 and high risk above 3 mg/L (James SK et al. 2003). Liuzzo et al. (Liuzzo et al. 1994) used a cut-off of 3 mg/l, based on the 90th percentile of a normal distribution. Dichotomized at this cut-off point, CRP identified patients at increased risk of in-hospital recurrent ischemic events. Biasucci et al. observed that levels of CRP >3 mg/l at discharge distinguished those patients at higher risk of cardiovascular events. However the majority of studies with CRP assessed at entry for ACS have found 10 mg/L as an optimal cut-off point for future events, in particular death. Mueller et al. (Mueller et al. Circulation 2002) found that a serum CRP > 10 mg/l was associated with a higher risk of death in patients with NSTE-ACS despite aggressive management with early invasive therapy. More recently, Scirica et al. (Scirica BM et al. 2007) found that in patients with UA/NSTEMI, CRP levels >3 mg/L were associated with increased 10-month mortality, whereas in STEMI a relationship with mortality was seen at CRP levels >10 mg/L and therefore they suggested that CRP measurement should be performed early after presentation and index diagnosis-specific cutpoints should be used. The American Heart Association/Centers for Disease Control and Prevention (AHA/CDC) have defined specific cut-off points for clinical interpretation: CRP concentrations <1 mg/l are considered low, 1–3 mg/l are average and >3 mg/l indicate high relative risk (Pearson TA et al. 2003). A CRP concentration >10 mg/l appears to be the optimal cut-off point (when applied during hospitalization) for prediction of new AMI and death in secondary prevention (James SK et al. 2003).

Available data support the use of CRP in conjunction with troponin and other traditional clinical tools as a prognostic marker in patients with ACS. Studies from Ridker’s group (Ridker PM et al. 2002) demonstrate that CRP may give important information above those predicted by cholesterol levels in subjects at intermediate risk. Data from PROVEIT-TIMI22 (Ridker PM et al. 2005) have shown that optimal levels of LDL cholesterol don’t give optimal protection, unless CRP is also lowered below 2 mg/L. Accordingly, the AHA/CDC scientific statement on the use of markers of inflammation in cardiovascular disease in clinical practise includes recommendation for the use of CRP to identify patients at higher absolute risk of recurrent cardiovascular events and candidates for more aggressive monitoring and therapy, although the results of such strategy are not established. However, because CRP is closely but not specifically related to the presence of the inflammatory process underlying ACS, research activities have focused to the identification of more specific markers of vascular inflammation.

The relative and independent role of CRP in ACS in comparison to troponin and natriuretic peptides has been widely assessed and discussed. In 2003 Centre for Disease Control/American Heart Association (Pearson TA et al. 2003) disseminated a scientific statement on the use of CRP and other inflammatory markers, the consensus was that CRP may be useful to address and tailor intensive medical treatment after ACS, but that no definite evidence existed to suggest such strategy. More recently the National Academy of Clinical Biochemistry has published (NACB writing group 2007) the guidelines on clinical characteristics and Utilization of biochemical markers in ACS.

These guidelines consider cardiac troponin the best biomarker for initial evaluation and early risk stratification of ACS (both class IA), conversely natriuretic peptides (BNP and NT-proBNP) and CRP are not considered in the initial evaluation and have a class IIA (level of evidence A) recommendation for early risk stratification in ACS in addition to cardiac troponin, while their use a sole marker is discouraged. These guidelines appear well balanced and based upon the strong evidence in favor of troponin and, conversely, the lack of consistent data in favor of a clinical strategy based on natriuretic peptides and CRP.

## Marker of Plaque Destabilization and Rupture

### Placental growth factor (PIGF)

Placental growth factor (PIGF), a member of the vascular endothelial growth factor (VEGF) family, was shown to be profoundly upregulated in early and advanced atherosclerotic lesions (Luttun A et al. 2002). PIGF is a 50 kDa platelet-derived protein consisting of 149 aminoacids. It exists in two forms, PIGF-1 and PIGF-2, differing only for an aminoacid. Originally identified in the placenta (Maglione D et al. 1991), PIGF mRNA is expressed by various tissues, including thyroid and lung (Tjwa M et al. 2003). PIGF functions are incompletely understood. It might have a role in the first phases of the inflammatory process through stimulation of vascular smooth muscle growth, recruitment of macrophages into atherosclerotic lesions, upregulation of tumor necrosis factor-α (TNF-α) and monocyte chemoattractant protein-1 production by macrophages (Heeschen C et al. 2004). Besides, it enhances production of Tissue Factor and stimulates pathological angiogenesis (Autiero M et al. 2003). Interestingly, experimental PIGF inhibition, by blocking its receptor Flt-1, suppresses both atherosclerotic plaque growth and vulnerability via inhibition of inflammatory cell infiltration (Pearson TA et al. 2003). All these data suggest that PIGF may act as a primary inflammatory instigator of atherosclerotic plaque instability and therefore, inhibition of its effects may represent a new therapeutic target.

In 2004 Heeschen et al. (Heeschen C et al. 2004) investigated the potential role of PIGF for assessing risk of death or non-fatal myocardial infarction in the 30 days after index presentation. They studied 1173 patients divided into two cohorts: one having angiographically confirmed ACS (n = 547 enrolled in the CAPTURE trial) and the other presenting to the Emergency Department (ED) with chest pain (n = 626 enrolled in Germany). In order to avoid the possibly confounding effect of antiplatelet therapy only patients of the CAPTURE placebo arm were included in the assessment of PIGF. In the CAPTURE cohort, 40.8% patients were found to have increased PIGF concentrations and were found to have a markedly increased risk of adverse events at 30 days; also in the other cohort, PIGF was a predictor of increase in risk. These data suggest that PIGF blood levels may represent a novel, powerful, independent, prognostic determinant of clinical outcome in patients with ACS. Intriguingly, elevated PIGF levels identified not only patients with ACS presenting with acute chest pain, but also patients with an increased risk of recurrent instability after discharge. The predictive value of PIGF levels is independent of myocardial necrosis, as evidenced by elevated troponin levels (Heeschen C et al. 1999), as well as platelet activation, as evidenced by elevation of sCD40L (Heeschen C et al. 2003a).

In summary, although large trials are still needed to obtain PIGF validation and commercialization, it appears to have great potential as an independent biomarker for plaque disruption, ischemia and thrombosis and also as a novel anti-inflammatory therapeutic target in patients with CAD.

### Myeloperoxidase (MPO)

Myeloperoxidase (MPO) is a hemoprotein (molecular mass ~140 kDa) composed of a pair of heavy and light chains. It is an enzyme that catalyzes the conversion of chloride and hydrogen peroxide to hypo-chlorite and has the optimum of activity at acid pH. It is stored in azurophilic granules of polymorpho-nuclear neutrophils and macrophages and is released into extracellular fluid in the setting of inflammatory process. MPO catalyzes activation reaction of several lysosomial proteins released as proenzymes from granulocytes (Klebanoff SJ, 1999) and has been implicated in the oxidation of lipids contained within Low Density Lipoprotein (LDL) (Holvoet P, 1998). In addition, MPO consumes endothelial-derived NO, thereby reducing NO bioavailability and impairing its vasodilatatory and antinflammatory functions. Furthermore, MPO has been showed to activate metalloproteinases and promote destabilization and rupture of the atherosclerotic plaque surface. The observation that MPO is involved in oxidative stress and inflammation has been a leading factor to study MPO as a possible marker of plaque instability. Oxidative stress and inflammation play a pivotal role in the pathogenesis of the destabilization of CAD leading to ACS: macrophages and neutrophils are implicated in the transformation of stable coronary artery plaques to unstable lesions and are found in high levels in the culprit lesions of patients with ACS (Takahiko N et al. 2002). They act by degrading the collagen layer that protects atheromas from erosion or abrupt rupture and in this setting, MPO has a pivotal role. In 1996, Biasucci et al. (Biasucci LM et al. 1996) observed that circulating neutrophils in patients with AMI and UA have a low MPO content, compared with those with chronic stable angina and variant angina. This is indicative of a significant release of MPO from neutrophils related to their activation. Although MPO participates in the inflammatory process of ACS, neutrophil activation is apparently not induced by ischemia. In 2001, Zhang et al. (Zhang R et al. 2001), showed that blood and leukocyte myeloperoxidase activities were higher in patients with CAD than angiographically verified normal controls, and that these increased activities were significantly associated with presence of CAD ( odds ratio, 11.9; 95% confidence interval, 5.5–25.5). Results were independent of patient’s age, sex, hypertension, smoking, diabetes status, LDL concentration, leukocyte count, Framingham Global Risk Score. Buffon et al. (Buffon A et al. 2002) have enrolled 65 patients who underwent cardiac catheterization with coronary sinus sampling and have observed that increases in MPO correlated with value of CRP in patients with ACS. The potential role of MPO as a marker for risk stratification in patients with ACS was examined in recent studies. Brennan et al. (Brennan ML et al. 2003) have enrolled 604 patients admitted to the ED with chest pain. They have investigated the correlation between risk of major cardiac events at 30 days and 6 months and increase in MPO concentration. The result was a progressive increase in odds ratios for major cardiac events with each quartile of MPO concentration. Even in the absence of myocardial necrosis, baseline measurements of MPO significantly enhanced the identification of patients at risk. Intriguingly, MPO was independent of both troponin and CRP levels. MPO predicted adverse outcome independently of sCD40L. This may imply that neutrophil activation represents an adjunct patho-physiological event in ACS that is distinctly different from platelet activation. In the CAPTURE trial (Baldus S et al. 2003), MPO mass concentration was measured in 1090 patients with diagnosis of ACS and the death and MI were determined at 6 months of follow up. The result was that MPO and the other markers studied (cTnT, soluble cd40L, CRP and vascular endothelial growth factor) were independent predictors of adverse cardiac events. Using a cut-off of 350 μg/l for MPO in patients with ACS, adjusted hazard ratio was 2.25 (95% CI 1.32–3.82) and considering only patients with undectable cardiac troponin the hazard ratio was 7.48 (95% CI 1.98–28.29). Furthermore, Cavusoglu et al. (Cavusoglu E et al. 2007) have investigated the long-term prognostic significance of baseline MPO levels in a well-characterized cohort of 193 men with ACS and have demonstrate that baseline MPO levels independently predict MI at 2 years in patients with ACS. Similar data were also reported by Mocatta et al. (Mocatta TJ et al. 2007). Importantly Mocatta et al. also showed that MPO prediction of future events is independent from NT-proBNP and LVEF values. These findings contrast with data from Apple et al. (Apple FS et al. 2007), who investigated correlation between multiple biomarkers and adverse outcomes rate in patients presenting to ED with symptoms suggestive of ACS. In this study MPO, sCD40L and MMP-9 levels were not independently associated with death, whereas patients with increased levels of PIGF, NT-proBNP, CRP, cTnI or decreased eGFR had higher mortality rate than patients with normal value of these biomarkers. Of note, however, patients with both cTnI and MPO raised levels had a mortality rate close to 40%.

In summary, observations from studies that have investigated MPO activity, demonstrated MPO is more than a marker of oxidative stress and not only a marker of plaque instability. These pathophysiological characteristics are very interesting but more studies are needed to assess sensitivity and specificity of this biomarker in particular in heterogenous populations.

### Pregnancy-associated plasma protein (PAPP-A)

Pregnancy-associated plasma protein (PAPP-A) is a high molecular mass (~200 kDa) glycoprotein typically measured during pregnancy for screening of Down syndrome (Wald NJ et al.1996). Physiologically, PAPP-A circulates in a hetero-tetrameric complex consisting of two PAPP-A subunits covalently bound with two subunits of the pro-form of eosinophil major basic protein (proMBP), its endogenous inhibitor (Oxvig C et al. 1993). PAPP-A has also been implicated in coronary plaque disruption (Lawrence JB et al. 1999). Preliminary results provide evidence that circulating PAPP-A during ACS is different from PAPP-A isolated from pregnancy sera (Quin QP et al. 2002). It is released during atherosclerotic plaque disruption in a homodimeric active form, uncomplexed with the inhibitor proform of eosinophil major basic protein (proMBP) contrary to the form present during pregnancy. Bayes-Genis et al. found abundant PAPP-A expression in unstable plaques but not in stable plaques from patients died of sudden cardiac death, mostly in the inflammatory shoulder region. They also described increased PAPP-A concentrations in the serum of patients with both UA and AMI, with PAPP-A levels >10 mIU/l identifying ACS patients with a sensitivity of 89% and a specificity of 81% (Bayes-Genis A et al. 2001).

PAPP-A blood levels seem to be an independent predictor of ischemic cardiac events and indicative of revascularization in patients with suspected MI but negative troponin: Lund J et al. found that PAPP-A plasma levels >2.9 mUI/l were associated with a risk of MI, death or revascularization 4.6-fold higher vs PAPP-A plasma levels <2.9 mUI/l (Lund J et al. 2003). Similar results were obtained by Heeschen et al. (Heeschen C et al. 2003b). They demonstrated the role of PAPP-A as independent marker of future cardiac events in ACS patients. However the overall correlation of PAPP-A with Catnip and CK-MB concentrations appears to be weak, indicating that increased PAPP-A cannot be attributed to myocardial necrosis. A significant association between PAPP-A and CRP concentration was shown in the first studies, but was not confirmed by subsequent papers. (Heeschen C et al. 2003b; Oxvig C et al. 1993).

Although preliminary studies suggest that PAPP-A may be useful to evaluate patients with ACS, additional investigations will be necessary for better acceptance of PAPP-A as an independent biomarker for cardiovascular risk in ACS. It is difficult to measure PAPP-A present in atherosclerotic plaques by immunoassays because they are designed to detect PAPP-A present in pregnancy sera. The kinetics of PAPP-A release and the corresponding optimal sampling protocols in ACS remain to be determined (Quin QP et al. 2002). Because preliminary findings showed that serum PAPP-A concentrations sensitively reflect changes in renal function and correlate with serum creatinine, the possible influence of renal function on PAPP-A concentrations should be also clarified (Fialova L et al. 2004).

### Metalloproteinase-9

Metalloproteinases (MMPs) are a class of 24 endopeptidases that are physiologic regulators of the extracellular matrix (Visse R and Nagase H, 2003). They are an expanding group of proteolytic enzymes that participate in numerous physiological and pathological processes including embryogenesis, connective tissue turn-over, healing, angiogenesis (Dabek J et al. 2007). Disturbances in matrix activity are observed in carcinogenesis, in some degenerative processes, and in inflammatory conditions, including atherogenesis. The role of matrix in the pathology of the cardiovascular system seems to be particularly important in two processes: (1) atherosclerotic plaque development and rupture (leading to an acute coronary event) and (2) post-infarction remodelling of myocardium, leading to heart failure. Human atherosclerotic lesions overexpress human interstitial collagenases, members of the matrix metalloproteinase family (Dollery CM and Libby P, 2006). Inflammatory stimuli augment the production of the interstitial collagenases MMP-1, MMP-13, MMP-8 from several cell types found in atherosclerotic plaques. Human atherosclerotic plaque also contains elevated levels of MMP-9 active form, an enzyme with gelatinolytic activity that can continue the catabolism of collagen cleaved by interstitial collagenases (Galis Z et al. 1994a). Inflammatory mediators regulate this enzyme, which can also degrade elastin ( Galis Z et al. 1994b). Recently, Fiotti et al. (Fiotti N et al. 2007) have studied patients with stable angina and patients with ACS undergoing PCI and have assessed MMP-2, MMP-9 and TIMP-1 expressions. They have found that MMP-9, but not TIMP-1 or MMP-2 expression is increased in plaques causing ACS. MMP-9 is localized in the plaque shoulder, the thinner area prone to rupture. So MMP-9 may be the most promising, among MMP, as marker.

Kai H et al. ( Kai H et al. 1998) measured MMP-9 concentration in ACS patients and stable angina patients compared with healthy controls. Patients with stable angina had a MMP-9 concentration similar to healthy controls; all patients with UA had elevated levels of MMP-9 at entry, that decreased gradually during hospitalization. Intriguing data from Inokubo’s group (Inokubo Y et al. 2001) showed a substantial gradient of both MMP-9 and tissue inhibitor of metalloproteinases 1 (TIMP-1) across the coronary sinus suggesting a local release/production of these molecules.

The potential role of MMP-9 as a marker for risk stratification of patients with ACS was examined by Blankenberg et al. (Blankenberg S et al. 2003): they studied 1127 patients with stable (n = 795) and unstable (n = 332) angina and found that MMP-9 values were related to future cardiovascular death. Its prognostic value was also maintained after correction for CRP, fibrinogen, IL-6, IL-18. Indeed, MMP production seemed to be important for reparative process of cardiac tissue after injury, although previous data showed that transgenic animals susceptible to ventricular rupture are protected by deletion of MMP-9 gene ( Heymas S et al. 1999).

Briefly, preliminary studies suggest that MMP-9 may be of value in evaluating ACS patients but additional investigations will be necessary for better acceptance of MMP-9 commercialization and clinical application.

## Markers of Ischemia-Necrosis

### Fatty acid binding proteins (FABPs)

FABPs are low-molecular-mass proteins that are abundant in the cytoplasm of tissues having active fatty acid metabolism, including the heart, striated muscle, liver and intestine. FABPs are the major vehicle for cytosolic transport of long-chain unesterified fatty acids (Ockner RK et al. 1972). Myocardium and skeletal muscle contain the same isoform of FABP, termed heart-type FABP (H-FABP), but the content of this protein in skeletal muscle is only 10% –30% of that found in cardiac muscle. H-FABP concentration in healthy donors is relatively low (2–6 μg/l) and it has a very good tissue/plasma ratio. Age, sex and circadian rhythm significantly influence H-FABP reference value. Probably because of their larger muscle mass, men have higher plasma H-FABP concentration than women. Because H-FABP has predominantly a renal clearance and renal function decreases with age, plasma H-FABP may increase with age (Tsuji R et al. 1993).

H-FABP appears in blood soon after the onset of infarction, so it has been proposed as an early marker for the MI diagnosis (Kleine AH et al. 1992). Its plasma concentration increase within 2–3 h after MI and return to the normal range within 12–24 h in individuals without renal impairment.

In general, H-FABP was found to perform better than or similar to myoglobin in diagnosis of ACS (Okamoto F et al. 2000), probably due to the higher cardiac tissue content of H-FABP compared with myoglobin. Seino et al. (Seino Y et al. 2004) compared blood rapid test for H-FABP with rapid cTnT test regarding diagnostic accuracy in cardiac ischemia: rapid H-FABP assay seemed to effectively exclude non-AMI patients within 3 h of onset. Therefore, H-FABP may be useful in the diagnostic assessment of ACS patients in ED, in combination with troponin, along with the electrocardiographic and clinical evaluation. Because of different amounts of H-FABP and myoglobin in myocardial and skeletal muscle tissue a low mioglobin/H-FABP ratio might have myocardial specificity. In fact, myoglobin/H-FABP ratio was found to be low (values in the range of 2–10) in plasma from patients with myocardial injury and high (values in the range of 20–70) in patients with skeletal muscle damage (Van Nieuwenhoven FA et al. 1995). However, strategies to improve detection of myocardial injury by calculating myoglobin/H-FABP ratio haven’t yielded a clear advantage over the measurement of H-FABP alone. Nakata et al. have shown that H-FABP has greater diagnostic value and sensitivity than cTnT, CKMB and myoglobin in patients with suspected ACS within 6 h from acute chest pain onset (Nakata T et al. 2003) and an early or sustained elevation in H-FABP could indicate unfavourable clinical outcome (Suzuki M et al. 2005), as confirmed by Nagahara (Nagahara D et al. 2006), too, who found a higher positive predictive value (84%–91%), a higher sensitivity (75%–77%) and a better negative predictive value (nearly 40%) for H-FABP vs cTnT, CK-MB and myoglobin. However, in these studies low sensitivity troponins were used, therefore reducing cTnT sensitivity for ACS. In a recent study O’Donoghue et al. (O’Donoghue et al. 2006), have evaluated H-FABP, Troponin and BNP levels of 2287 patients with ACS from the OPUS-TIMI 16 trial and have found that elevation of H-FABP is associated with an increased risk of death and major cardiac events at 10 months of follow up, independently from other established clinical risk predictors and biomarkers. Indeed, it provides incremental prognostic information regardless of baseline troponin or BNP status.

Because H-FABP rapidly return to the normal range within 24 h after AMI, it can be also used to assess a recurrent infarction within 10 h after first AMI, possibly missed by CK-MB, cTnT and cTnI evaluation because plasma concentration of these markers returns more slowly to reference values. H-FABP has a renal clearence, therefore impaired renal function potentially impact its clinical utility; however, data from de Groot et al. (de Groot et al. 1999) indicate that H-FABP, after correction for estimated renal function, can be applied successfully for infarct size estimation.

The rapid release of H-FABP can also be used for the detection of successful coronary reperfusion in patients with AMI (Suzuki M et al. 2005). Both plasma H-FABP and myoglobin levels were found to increase sharply after successful reperfusion, slowly after failed reperfusion.

Suzuki and coll. (Suzuki M et al. 2005) have recently showed that a positive H-FABP test is an independent predictor of adverse events within 30 days in patients with ACS. Increased plasma H-FABP concentration significantly correlate with cardiac events and mortality (Ishii J et al. 2003). Although H-FABP is generally considered a marker of myocardial necrosis, a recent study has indicated its additional potential utility as a marker of ischemia, also in absence of myocardial necrosis, therefore it could be useful for early identification of ACS in patients with chest pain of uncertain origin (Tambara K et al. 2004).

### Free fatty acids unbound to albumin (FFAu)

Free fatty acids unbound to albumin (FFAu) were also evaluated for early identification of cardiac ischemia. FFAs are localized in the cytoplasm, bound to fatty acid binding protein (FABP) and represent the primary metabolic sources for myocardium. During hypoxia and ischemia, elevated blood cathecolamines increase FFAu concentration through adipose lipolyses activation. Increased non-esterified fatty acids/FFA levels have damaging effects on heart tissue and have been associated with an increased incidence of ventricular dysrhytmias and death in patients with AMI.

Several investigators have preliminarily evaluated the sensitivity of this marker on admission to emergency room and have shown that FFAu elevation occurs before other, more traditional, markers of cardiac necrosis (Adams JE et al. 2002). In the TIMI II trial FFAu concentration were measured in 458 patients on admission and 50 min, 5 h and 8 h after the initiation of tissue Plasminogen Activator (tPA) treatment (Kleinfeld AM et al. 2002). Sensitivity of FFA was 91% at admission and 98% 50 min after tPA (cut-off 5 nmol/l); specificity, compared with healthy individuals and patients with non cardiovascular disease, was 93%. FFAu were increased in 100% of MI patients on admission, whereas only 22% of these patients had increased cTnI at presentation, indicative of earlier appearance of this analyte in the circulation before traditional markers of myocite necrosis (Apple FS et al. 2005).

FFAu have also been studied for risk stratification of ischemic heart disease (IHD). In the Paris Prospective Study I, increased plasma FFAu concentration were found to be an independent risk factor of sudden death, but not of fatal MI in a cohort of men without known ischemic cardiac disease (Jouven X et al. 2001). Pirro et al. (Pirro M et al. 2002) studied the relationship between circulating FFAu concentration and risk of IHD in 2130 men with insulin resistance syndrome and without IHD at enrollment. During a 5-year follow-up, 114 of these individuals developed IHD. After adjustment for nonlipid risk factors, increased circulating FFAu levels conferred a 2-fold increase of IHD risk (odds ratio, 2.1; *P* = 0.05). However, after adjustment for triglyceride concentrations, HDL-cholesterol, small and dense LDL, apolipoprotein B, and fasting plasma insulin, the relationship between plasma FFAu concentration and IHD development didn’t achieve statistical significance.

Pilz et al. (Pilz S et al. 2006) studied 3315 Caucasians from LURIC study to elucidate the relationship between FFAu and mortality in subjects undergoing coronary angiography at a median follow up of 5.38 years. This follow-up study demonstrated that high levels of FFAu may predict total and cardiovascular mortality and its predictive value for all-cause and cardiovascular mortality remains stable even after multivariable (CRP, creatinine, homocysteine, NT-proBNP) adjustments. These support the idea of a direct involvement of FFAu in atherosclerotic pathophysiological process.

In conclusion, current data, although limited, suggest that monitoring FFAu concentrations in patients presenting with ischemia symptoms may provide an early indication of cardiac ischemia. Additional studies are needed to fully evaluate the true potential of this biomarker.

### Ischemia modified albumin (IMA)

Ischemia modified albumin (IMA), measured by the albumin cobalt binding test (ACB), has been shown to be a marker of myocardial ischemia. A multicenter study (Christenson RH et al. 2001), involving 224 patients, who arrived at the ED within 3 h after symptoms onset, examined the ability of the ACB test to predict a positive or negative cTnI result within 6–24 h after presentation. All patients had a negative cTnI result at presentation. At the optimum cut-off for the ACB test, sensitivity and specificity were 70% and 80%, respectively, with a negative predictive value of 96%. In the study by Bhagavan et al. (Baghavan NV et al. 2003) the sensitivity and specificity for myocardial ischemia were 88% and 94%, respectively, and the positive and negative predictive values were 92% and 91%. Another study (Sinha MK et al. 2004) evaluated IMA in conjunction with EKG results and cTnT concentrations (>0.05 μg/L) in 208 patients presenting to the ED within 3 h of acute chest pain. In the whole patient group, sensitivity of IMA at presentation for an ischemic origin of chest pain was 82%, specificity was 46%, the negative predictive value was 59%, and the positive predictive value was 72%. IMA, EKG, and cTnT combined identified 95% of patients whose chest pain was attributable to ischemic heart disease.

The positive predictive value of the ACB assay seems to be too low for use in ruling in ischemia. At present, whether patients with negative EKG results and necrosis markers, i.e. cardiac troponins, and a positive IMA result might benefit from early triage and intervention according to stratified pre-test probabilities is not known. In clinical practice, this lack of information can potentially lead to overtreatment of low-risk patients with a positive result.

Recent findings suggest that IMA may be also useful in risk stratification of emergency chest pain patient (Pollack CV et al. 2003). Peacock et al. (Peacock et al. 2006) have performed a meta-analysis of IMA in ACS risk stratification. In the presence of a triple negative prediction test (nondiagnostic electrocardiogram, negative troponin, and negative IMA) they found that sensitivity and negative predictive value for ACS were 94.4% and 97.1% and, for long-term outcomes, were 89.2% and 94.5%, respectively. However, in the short term, in patients presenting with chest pain who have not yet experienced a serious cardiac event, IMA is a poor predictor of serious cardiac outcomes (death, myocardial infarction, congestive heart failure, serious arrhythmia, or refractory ischemic cardiac pain) (Worster A et al. 2005).

IMA appears to be indicative of oxidative stress and, therefore, might not be specific for cardiac ischemia. Increased IMA values are also found in patients with cancer, infections, end-stage renal disease, liver disease, and brain ischemia (Wu AH, 2003).

In summary, many questions remain unanswered regarding IMA and the ACB test. The highest expected benefit of the test would be to rule out ACS in low to moderate pre-test probability conditions with negative myocardial necrosis markers and a negative EKG. However, the test seems to have limited specificity, with many false positives. Additional information, including a better understanding of IMA kinetics in the first hours after ACS and an optimum diagnostic cut-off value assessment, is needed for clinical validation of this new assay.

## Conclusions

Increasing evidence suggests that the use of biomarkers, reflecting distinct pathophysiological features such as oxidative stress, vascular inflammation, platelet activation, plaque destabilization and rupture along with more sensitive biomarkers of necrosis, may improve the traditional diagnostic and risk stratification approach in order to apply tailored treatment. However, no available biomarker offers ideal properties such as very early raise, very high sensitivity and specificity, easy and cheap assay. These observations suggest a multi-marker strategy, employing patho-biologically different biomarkers, that might help significantly in the identification of the “vulnerable” patient at risk for CHD, as suggested by a recent National Institutes of Health (NIH) panel (Morrow DA et al. 2003). A multi-marker strategy, based on the use of a combination of two or more markers to enable the detection of myocardial infarction in patients who seek care early and late after symptom onset, could consent an early diagnosis and facilitate identification of patients standed for aggressive interventions. However there is no evidence that a strategy based on measurement of more than two markers (besides troponin and CRP or natriuretic peptides) might improve diagnostic and prognostic power of a two markers strategy. So far, indeed, consistent and reliable data on diagnostic and prognostic value of these markers are available only for Troponin, BNP and CRP. Further studies are need to establish the role of new biomarkers and to cross boundaries from research to clinical practice.
